# miR-22 is down-regulated in esophageal squamous cell carcinoma and inhibits cell migration and invasion

**DOI:** 10.1186/s12935-014-0138-0

**Published:** 2014-12-12

**Authors:** Chao Yang, Siqing Ning, Zhaoyuan Li, Xiaomin Qin, Wei Xu

**Affiliations:** Department of Oncology, Xiangyang Central Hospital, Affiliated Hospital of Hubei University of Arts and Science, Xiangyang, 441000 China; Department of Dermatology, Xiangyang Central Hospital, Affiliated Hospital of Hubei University of Arts and Science, 136 Jingzhou Street, Xiangyang, 441000 China

**Keywords:** miR-22, Esophageal squamous cell carcinoma, Invasion, Migration

## Abstract

**Background:**

Esophageal squamous cell carcinoma (ESCC) is one of the most common and deadly forms of cancer. Despite advances in the diagnosis and treatment of this cancer, the survival rate at five years is poor. Lately, miR-22 is identified as a tumor-suppressing microRNA in many human cancers. However, the specific function of miR-22 in ESCC is unclear at this point.

**Methods:**

We first measured miR-22 expression level in 30 paired of ESCC and matched normal tissues, ESCC cell lines by real-time quantitative RT-PCR. Invasion assay, MTT proliferation assay and wound-healing assay were performed to test the invasion and proliferation of ESCC cell after overexpression of miR-22.

**Results:**

We found that the expression of miR-22 in ESCC tissues and cell lines were much lower than that in normal control, respectively. The expression of miR-22 was inversely correlated with ESCC metastatic ability. Furthermore, transfection of miR-22 expression plasmid could significantly inhibit the cell proliferation, migration and invasion in Eca109 and Kyse410 ESCC cell lines.

**Conclusions:**

Our findings suggest that miR-22 act as tumor suppressor and inhibiting ESCC cell migration and invasion. The findings of this study contribute to the current understanding of the functions of miR-22 in ESCC.

## Background

Esophageal cancer, one of the most common malignant tumors, is the eighth most common cancer and the sixth most common causes of cancer mortality in the world [1,2]. Esophageal cancer can be divided into two main forms: esophageal squamous cell carcinoma (ESCC) and adenocarcinoma [3]. Adenocarcinoma is common in western countries but ESCC is predominant in East Asia, especially in China [1,4]. Although advances have been made in the treatment of ESCC, including surgery, chemotherapy, radiation or a combination of these options, the prognosis of ESCC patients remains very poor, which the overall 5-year survival rate of patient after surgery is only about 14-22% [5]. In this way, there is a great need to discover more biomarkers and therapeutic targets for ESCC.

MicroRNAs (miRNAs), which encode small non-coding RNAs of approximately 22 nucleotides, are now recognized as a very large gene family. miRNAs can be classified as oncogenes or as tumor suppressors, and by targeting various transcripts they participate in diverse processes, including proliferation, apoptosis, metabolism, and cellular differentiation, etc. [6]. miR-22 is a 22-nt non-coding RNA and was originally identified in HeLa cells as a tumor-suppressing miRNA. Subsequently, miR-22 was identified to be ubiquitously expressed in a variety of tissues [7]. Recently, several targets of miR-22 were reported to mediate its tumorsuppressive effect, such as tumor-suppressive PTEN, Max genes, p21, Sp1, CD147 and oncogene c-Myc expression, etc. [7-11]. However, the expression and role of miR-22 in ESCC have not yet been clarified.

In the current study, we validated the differential expression of miR-22 in ESCC and investigated the function of miR-22 in migration and invasion of ESCC cancer cells. miR-22 might act as a tumor suppressor and serve as a potential therapeutic target in ESCC. To the best of our knowledge, this is the first study to examine the expression and mechanism of miR-22 in ESCC migration and invasion.

## Results

### The expression of miR-22 is down-regulated in ESCC tissues and cell lines

**Figure 1:**
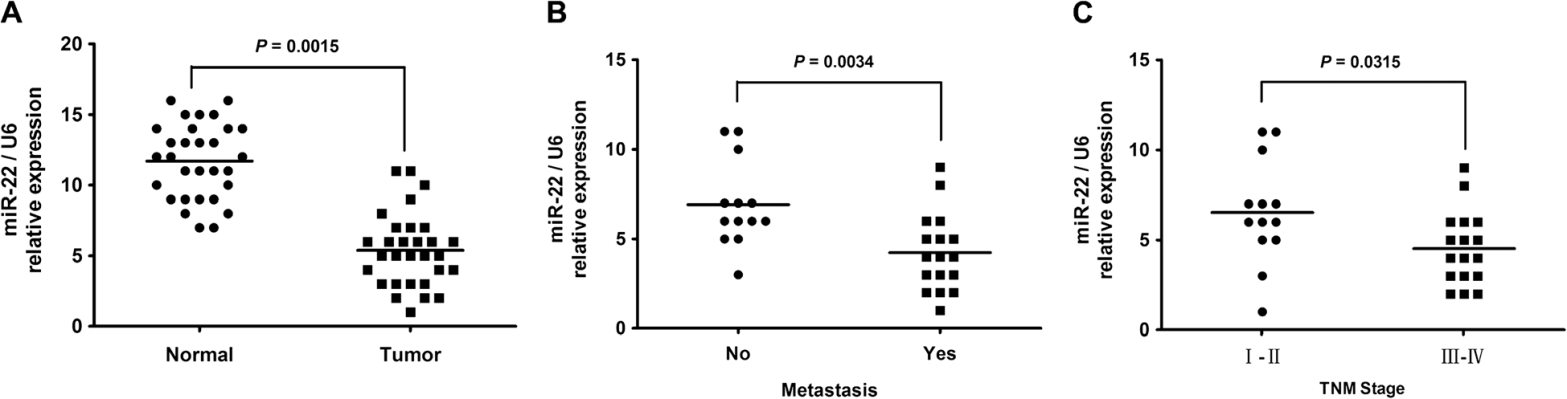
**miR-22 is down-expressed in ESCC tissues. (A)** The relative levels of miR-22 in thirty paired of ESCC samples were measured by real-time quantitative RT–PCR, and the U6 small nuclear RNA was used as an internal control. Student’s *t* test was used to analyze the significant differences between the tumor and normal tissues. **(B)** and **(C)** The expression of miR-22 in metastasis tumors and advanced histologic grades. Student’s *t*-test was used to analyze the significant differences.

The expression levels of miR-22 were first evaluated in thirty paired of ESCC and normal tissues by real time RT-PCR. As showed in Figure 1A, we found that tumor tissues showed aberrant downregulation of miR-22 compared with adjacent non-tumor tissues (*P* = 0.0015). We also confirmed that miR-22 expression was significantly reduced in metastasis tumors (*P* = 0.0034) and advanced histologic grades (*P* = 0.0315, Figure 1B,C). Therefore, we conclude that miR-22 expression is significantly down-regulated in ESCC at mRNA levels in a manner negatively associated with aggressive tumor behaviors.

**Figure 2:**
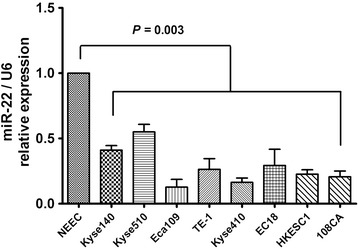
**The relative expression levels of miR-22 in the eight ESCC cell lines and one normal esophageal epithelial cell lines.** Student’s *t*-test was used to analyze the significant differences.

We also detected the miR-22 expression in ESCC and normal esophageal epithelial cell lines. We performed real-time RT-PCR on a panel of eight ESCC and one normal cell line. As showed in Figure 2, miR-22 levels of all cancer cell lines were lower than that of normal esophageal epithelial cells (NEEC) (*P* = 0.003). miR-22 expression in Eca109 and Kyse410 cells was relatively low. These results have increased our knowledge of the expression profile of miR-22 in tumor types and this specific expression mode indicates that miR-22 might play an important role in ESCC cancer progression.

### Over-expression of miR-22 inhibits ESCC cell proliferation

**Figure 3:**
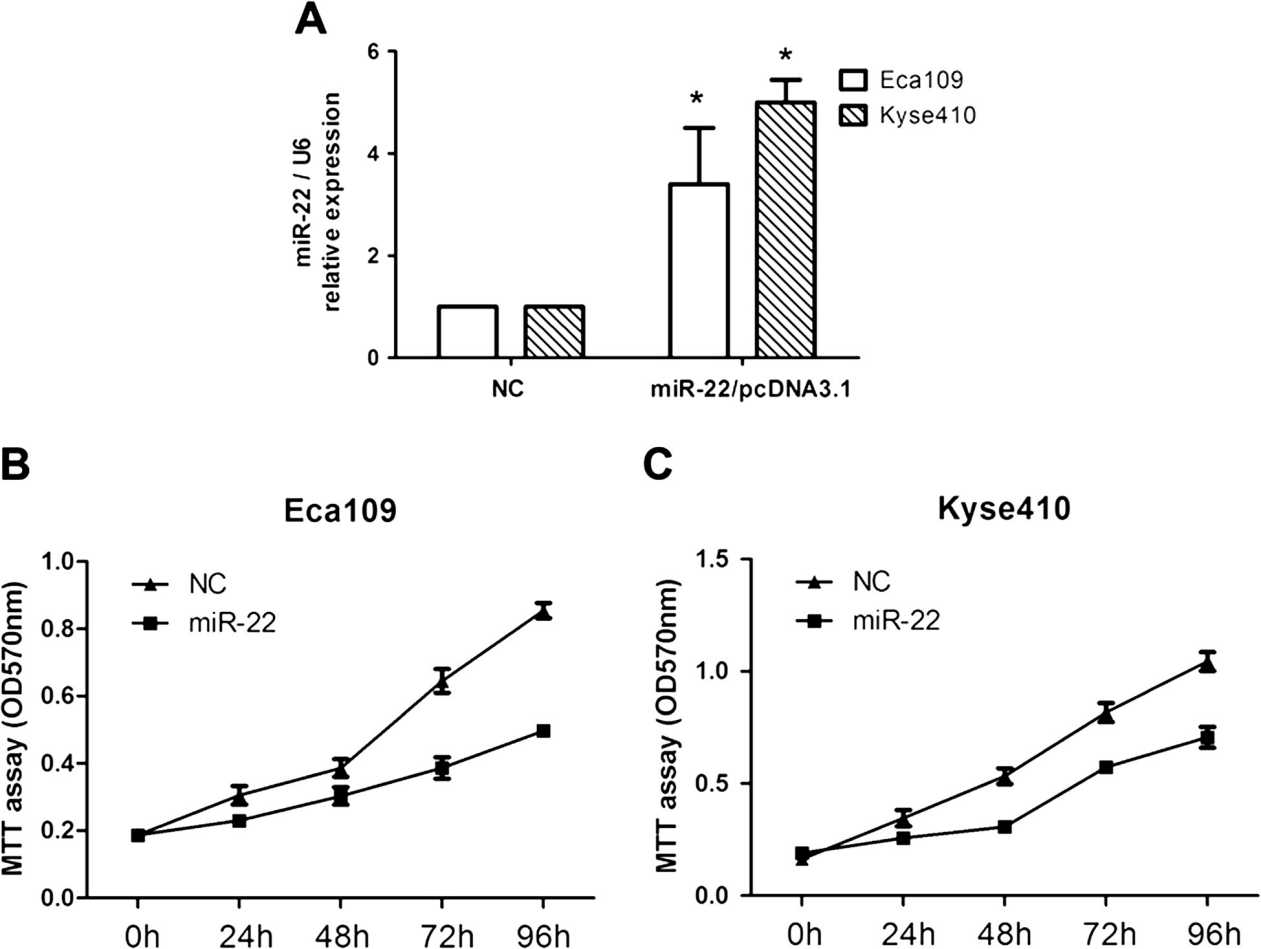
**Overexpression of miR-22 inhibits proliferation of ESCC cell lines. (A)** Transfection of miR-22 expression plasmid to Eca109 and Kyse410 increases the expression of miR-22 detected by real-time quantitative RT-PCR. *P < 0.05, by one-way ANOVA. **(B)** and **(C)** Cell proliferation of these cells transfected as in A was measured in the indicated time periods using the MTT assay.

Based on the above results, we detected whether miR-22 can change the capacity of ESCC cells for proliferation. We transfected Eca109 and Kyse410 cells with miR-22 expression vector or pcDNA3.1 control, and then evaluated the cell growth rate. As expected, transfection of miR-22 expression plasmid into Eca109 and Kyse410 cells resulted in substantial increase of miR-22 expression compared with negative control (NC) transfected cells (Figure 3A, *P* = 0.023). We performed MTT cell proliferation assays with transfected miR-22 in Eca109 and Kyse410 cells. Our results showed that restoration of miR-22 expression suppressed cell proliferation in both of the ESCC cell lines (Figure 3B and C).

### miR-22 inhibits ESCC cell migration and invasion

**Figure 4:**
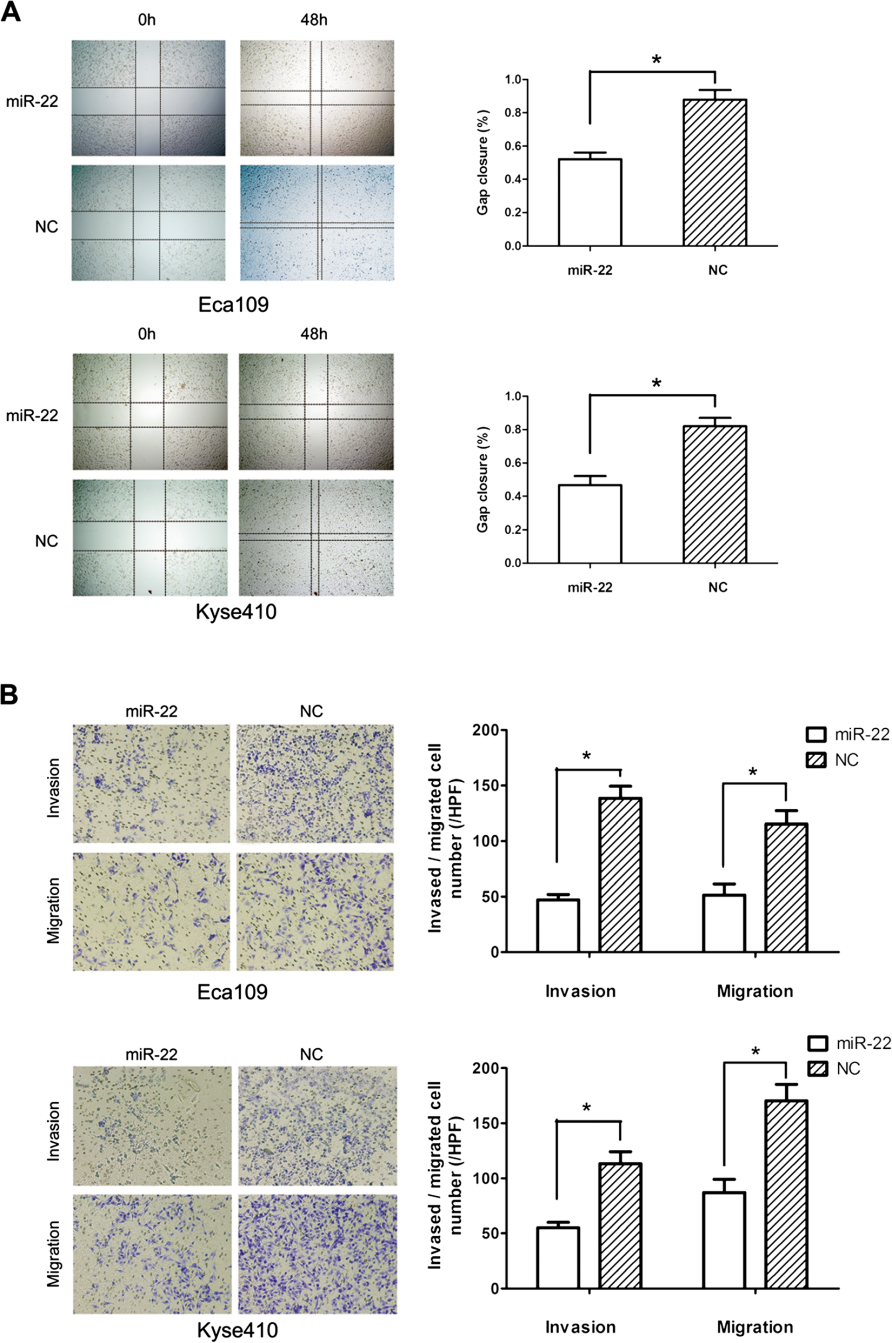
**Overexpression of miR-22 inhibits migration and invasion of ESCC cell lines. (A)** Overexpression of miR-22 presented a slower closing of scratch wound, compared with miRNA negative control, at 48 h after transfection in Eca109 and Kyse410 cells. **(B)** The inhibitory effect of miR-22 toward the invasion and migration of Eca109 and Kyse410 cells. *P < 0.05, by one-way ANOVA.

In addition to cell growth inhibition, the effect of miR-22 on tumor migration and invasion was also addressed in this study. The wound-healing assay showed that Eca109 and Kyse410 cells with miR-22 overexpression presented a slower closing of scratch wound, compared with the negative controls (Figure 4A, *P* = 0.019 and *P* = 0.021). Moreover, the cell migration and invasion assay showed that miR-22 restoration resulted in reduced migration rate and invasion rate of Eca109 and Kyse410 cells compared with the control (Figure 4B, upper *P* = 0.009 and *P* = 0.011, lower *P* = 0.027 and *P* = 0.029). Our results indicate that miR-22 served as a tumor suppressor miRNA and contributed to inhibition of migration and invasion of ESCC cells.

## Discussion

In our study, we demonstrated that miR-22 expression is decreased in human ESCC tissues and cell lines compared with matching adjacent non-tumoral tissue and normal cell lines. The down-expression of miR-22 correlates with ESCC metastatic ability. We also discovered that miR-22 suppress ESCC cell migration and invasion. The identification of miR-22 as an essential regulator of tumor cell migration and invasion *in vitro* emphasizes an essential role of this miR-22 in mediating ESCC oncogenesis and tumor behavior.

ESCC is the fourth most frequently diagnosed cancer and the fourth leading cause of cancer death in China [12]. Surgical resection is still as the mainstay strategy employed for operable ESCC. Despite the great advances has been achieved in multimodal therapy, its five-year survival rate remains unsatisfactory [5,13]. Discovering suitable biomarkers will probably be a key to monitoring cancer recurrence or screening high risk population of ESCC, giving information on the need for adjuvant or neoadjuvant therapy.

miRNAs have been demonstrated to have close relationship with ESCC. miR-22, originally identified in HeLa cells, has been found to be overexpressed in prostate cancer, but down-regulated in breast cancer, cholangiocarcinoma, multiple myeloma, and hepatocellular carcinoma [14]. In this study, we demonstrated that miR-22 expression is decreased in human ESCC tissues and cell lines compared with matching adjacent normal tissues and cell lines. Restoration of miR-22 in Eca109 and Kyse410 cells significantly inhibited cellular migration and invasion capability. Taken together, our results suggest that miR-22 as a tumor suppressor plays a role in the metastasis and progression of ESCC.

Metastasis is a key step of tumor progression in ESCC, which means a poor prognosis [15]. Metastasis is a series of sequential events, including detachment, migration, local invasion, angiogenesis, extravasation, survival in the circulatory system, extravasation, and regrowth in different organs [16,17]. Several miRNAs can modulate tumor metastasis [18]. The identification of miR-22 as an important regulator of tumor cell migration and invasion *in vitro* emphasizes an essential role of this miRNA in mediating ESCC oncogenesis and tumor behavior.

## Conclusions

In summary, the present study provides evidence to support that miR-22, a microRNA downexpressed in ESCC, inhibits cell migration and invasion of ESCC cells *in vitro*. These findings imply that miR-22 might be a suitable candidate for anticancer therapy.

## Methods

### ESCC tissues collection

Thirty paired tissue specimens of ESCC and matched normal tissues were obtained from Department of General Surgery in Xiangyang Central Hospital. The matched "normal tissue" was obtained from a 5 centimeter distance from the tumor margin, which were further confirmed by pathologist that they do not have tumor cells. All of the tissues were obtained at the time of surgery and immediately stored in liquid nitrogen until use. All individuals provided written informed consent, and the study was approved by the Ethics Committee of Xiangyang Central Hospital.

### Cell culture

Primary culture of normal esophageal epithelial cells (NEEC) was established from fresh specimens of the adjacent noncancerous esophageal tissue, which is over 5 cm from the cancerous tissue, according to a previous report [19,20]. The ESCC cell lines, including Kyse140, Kyse510, Eca109, TE-1, Kyse410, EC18, HKESC1 and 108CA, were grown in the Dulbecco’s modified Eagle’s medium (DMEM, Invitrogen, Carlsbad, CA, USA) supplemented with 10% fetal bovine serum (FBS) (HyClone, Logan, UT, USA) at 37°C in a humidified atmosphere of 5% CO2.

### Real-time quantitative RT-PCR

Total cellular RNA was extracted using the Trizol reagent (Invitrogen, Carlsbad, CA, USA) according to the manufacturer’s instruction. Briefly, about 100 mg ESCC or adjacent tissues in the liquid nitrogen were preserved in 2 mL Trizol. Then, the tissues were fully cracked using the homogenizer. The supernatants were obtained after 12000 × g, 4°C, centrifugation 5 minutes. Total RNAs were isolated from the supernatants of these samples following the manufacturer’s instructions. Real time PCR was performed using SYBR Premix Ex Taq™ II Kit (TaKaRa) according to the manufacturer’s protocol on an MX3005P QPCR system (Stratagene, La Jolla, CA, USA). The U6 small nuclear RNA was used as internal control. All of the reactions were run in triplicate. The delta-Ct method [21,22] for relative quantification of gene expression was used to determine miRNAs expression levels. Forward and reverse primers for miR-22, u6 snRNA, were 5’-AAG CTG CCA GTT GAA GAA CTG TA-3’ and Universal Primer (Qiagen), 5’-CTC GCT TCG GCA GCA CA-3’ and 5’-AAC GCT TCA CGA ATT TGC GT-3’, respectively. All primers above were synthesized by Shanghai Sangon Biological Engineering Technology and Services Co., Ltd (Shanghai, China).

### Plasmid construction

For the expression of miR-22, genomic fragment of Homo sapiens miR-22 precursor was amplified by PCR using the primer pairs: 5’- GGG GGA TCC CTG GGG CAG GAC CCT -3’ and 5’- GGG GAA TTC AAC GTA TCA TCC ACC C -3’ [23]. The PCR product was cloned into pcDNA3.1 (Invitrogen, Carlsbad, CA, USA) named as pcDNA3.1-miR-22. Lipofectamine 2000 (Invitrogen) was used for DNA plasmid transfection.

### Cell proliferation assay

Cells were plated in sextuplicate in 96-well plates (2 × 10^3^ per well) in 100 μL complete medium and allowed to attach overnight. 3-(4,5-Dimethyl-2-thiazolyl)-2,5-diphenyl-2H-tetrazolium bromide (MTT) (20 μL at 5 mg/mL; Sigma, St. Louis, MO) was added every 24 h and incubated for 4 h. The supernatant was discarded, the precipitate was dissolved in 200 μL dimethyl sulfoxide (DMSO), and plates were read with a microplate reader at 570 nm [14].

### Wound-healing assay

The wound-healing assay was used to evaluate the tumor cell motility capacity. Briefly, 1 × 10^6^ cells were seeded in six-well plates, cultured overnight, and transfected with miR-22 and control, respectively. When the culture had reached nearly 90% confluency, the cell layer was scratched with a sterile plastic tip and then washed with culture medium twice and cultured again for up to 48 or 72 h with serum-reduced medium containing 1% FBS. At different time points, photographic images of the plates were acquired under a microscope and the data were summarized based on sextuple assays for each experiment.

### *In vitro* invasion assay and migration assay

MilliCell (12 mm diameter with 8 μm pores) chambers (Millipore, Bedford, MA, USA) were pre-coated with Matrigel (BD, Bedford, MA, USA) on the upper side. A total of 1 × 10^5^ serum-starved gastric cancer cells were added to the upper compartment in medium supplemented with 0.1% serum, and the chambers were placed into 24-well plates with medium containing 10% serum. After 24 h at 37°C, invaded cells on the lower membrane surface were fixed and stained with 0.1% crystal violet. Invasive activity was quantified by counting nine high-power fields (HPFs, 400×) per chamber. Mean values were obtained from at least three individual chambers for each experimental point per assay. The migration assay is the same with invasion assay excepting no Matrigel was used and the permeating time for cells was 12 hours.

### Statistical analysis

All statistical analyses were performed using the SPSS 16.0 statistical software package (SPSS, Chicago, IL, USA). The significance of the data was determined using Student’s *t* test. All the statistical tests were two-sided, and a *P* value < 0.05 was considered significant.

## Abbreviations

ESCC: Esophageal squamous cell carcinoma
miRNAs: MicroRNAs
NEEC: normal esophageal epithelial cells
RT-PCR: Reverse transcription-polymerase chain reaction
MTT: 3-(4,5-Dimethyl-2-thiazolyl)-2,5-diphenyl-2H-tetrazolium bromide
